# LipF increases rifampicin and streptomycin sensitivity in a *Mycobacterium tuberculosis* surrogate

**DOI:** 10.1186/s12866-020-01802-x

**Published:** 2020-05-25

**Authors:** Ana Leticia Arriaga-Guerrero, Carlos E. Hernández-Luna, Joyce Rigal-Leal, Rene J. Robles-González, Laura Adiene González-Escalante, Beatriz Silva-Ramírez, Roberto Mercado-Hernández, Javier Vargas-Villarreal, Mario Bermúdez de León, Katia Peñuelas-Urquides

**Affiliations:** 1 Departamento de Biología Molecular, Centro de Investigación Biomédica del Noreste, Instituto Mexicano del Seguro Social, Monterrey, Nuevo León México; 2 Facultad de Ciencias Biológicas, Universidad Autónoma de Nuevo León, San Nicolás de los Garza, Nuevo León México; 3 Departamento de Inmunogenética, Centro de Investigación Biomédica del Noreste, Instituto Mexicano del Seguro Social, Monterrey, Nuevo León México; 4 Departamento de Biología Celular, Centro de Investigación Biomédica del Noreste, Instituto Mexicano del Seguro Social, Monterrey, Nuevo León México

**Keywords:** *Mycobacterium*, Tuberculosis, Rifampicin-resistance, Streptomycin-resistance, LipF, lipases

## Abstract

**Background:**

Mortality due to tuberculosis (TB) has increased due to the development of drug resistance, the mechanisms of which have not been fully elucidated. Our research group identified a low expression of *lipF* gene in *Mycobacterium tuberculosis* clinical isolates with drug resistance. The aim of this work was to evaluate the effect of lipase F (LipF) expression on mycobacterial drug resistance.

**Results:**

The effects of expressing *lipF* from *Mycobacterium tuberculosis* in *Mycobacterium smegmatis* on resistance to antituberculosis drugs were determined with resazurin microtiter assay plate and growth kinetics. Functionality of ectopic LipF was confirmed. LipF expression reduced the rifampicin (RIF) and streptomycin (STR) minimum inhibitory concentration (MIC) from 3.12 μg/mL to 1.6 μg/mL and 0.25 μg/mL to 0.06 μg/mL respectively, moreover a reduced *M. smegmatis* growth in presence of RIF and STR compared with that of a control strain without LipF expression (*p* < 0.05 and *p* < 0.01) was shown.

**Conclusions:**

LipF expression was associated with increased RIF and STR sensitivity in mycobacteria. Reduced LipF expression may contribute to the development of RIF and STR resistance in *Mycobacterium* species. Our findings provide information pertinent to understanding mycobacterial drug resistance mechanisms.

## Background

*Mycobacterium tuberculosis* is the main causative agent of tuberculosis (TB), which is the leading cause of mortality due to infection worldwide. In 2017, there was an estimated of 10 million TB cases and 1.3 million deaths [1]. The first antibiotic discovered to treat tuberculosis in 1947 was streptomycin (STR) [2], this drug acts inhibiting protein synthesis through 30S ribosomal subunit inhibition [3]. For many years this drug was used in monotherapy in TB treatment therefore high drug-resistance levels appeared and the incorporation of different antibiotics to the treatment scheme became necessary [4]. STR use is recommended as part of the second-line treatment regimen and only when amikacin is not available or its resistance had been confirmed [5]. Nowadays, the standard TB treatment includes antimicrobial drugs such as rifampicin (RIF), isoniazid (INH), pyrazinamide (PZA), and ethambutol (EMB) [1]. RIF is a semisynthetic molecule produced in *Streptomyces mediterranei* with broad spectrum antibacterial activity. Its mechanism of action consists in the inhibition of RNA polymerase activity by forming a stable complex with it [6, 7]. Currently, RIF is considered to be the most effective first-line anti-TB drug and when administered with PZA the treatment regimen diminished to 6 months [8]. *M. tuberculosis* resistant to RIF and INH has become a serious problem. TB that is resistant to both drugs is defined as multidrug resistant (MDR)-TB [9]. Currently the TB epidemic is further exacerbated by the existence of MDR-TB. In 2017, there were approximately 558,000 new MDR-TB cases worldwide [1, 10]. Deficient treatment adherence by patients leads to selection pressure for drug-resistant (DR)-TB strains. The emergence and spread of drug resistance pathogens, particularly MDR-TB strains, pose a serious threat to human health worldwide [11]. Horizontal gene transfer has not been reported in *M. tuberculosis*, and MDR-TB has been generally associated with mutations. However mutations have not been identified in some MDR strains, which suggests that other mechanisms could be involved [12, 13]. A clinical MDR *M. tuberculosis* isolate was reported to have differential gene expression compared with that in the pansensitive H37Rv strain. Notably, the MDR strain had *lipF* (Rv3487c) gene down-regulated [14]. This gene encodes for a lipase with phospholipase C and carboxylesterase activities and has particularly high activity with four-carbon para-nitrophenyl (pNP)-derivate ester substrates [15, 16]. Recently, lipases have been implicated in drug sensitivity and resistance [17, 18]. In a recent study of 24 clinical isolates of *M. tuberculosis* with varying drug resistance profiles and genetic backgrounds, *lipF* expression was found to be reduced in ~ 90% of these resistance strains compared with that in the pansensitive reference strain H37Rv [19]. Although lipase F has been studied in *M. tuberculosis* virulence [16, 20, 21]; its role in drug resistance has not been addressed. Therefore, the aim of the present work was to evaluate the effect of *lipF* expression on drug resistance in a *M. tuberculosis* surrogate*.*

## Results

### Nucleotide sequence comparison between pansensitive and MDR strains

**Fig. 1:**
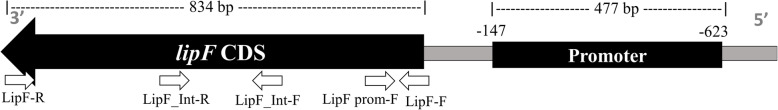
Genomic organization of *lipF* in *M. tuberculosis*. Promoter and coding sequence (CDS) are indicated. Coordinates are relative to the translation start site. Small arrows represent the primers used in cloning and in sequencing assays

Nucleotide sequence analysis were performed on the promoter and coding sequences of *lipF* to determine whether differential expression between the pansensitive H37Rv strain and the MDR CIBIN:UMF:15:99 clinical isolate, previously reported [14], could be due to mutations (Fig. 1). No sequence differences were found in the *lipF* promoter (477 bp), coding sequence (834 bp), or intergenic region (147 bp) between the two strains [Additional file 1], suggesting that the observed differential expression could involve other unknown regulation mechanisms.

### LipF expression in *Mycobacterium smegmatis*

**Fig. 2:**
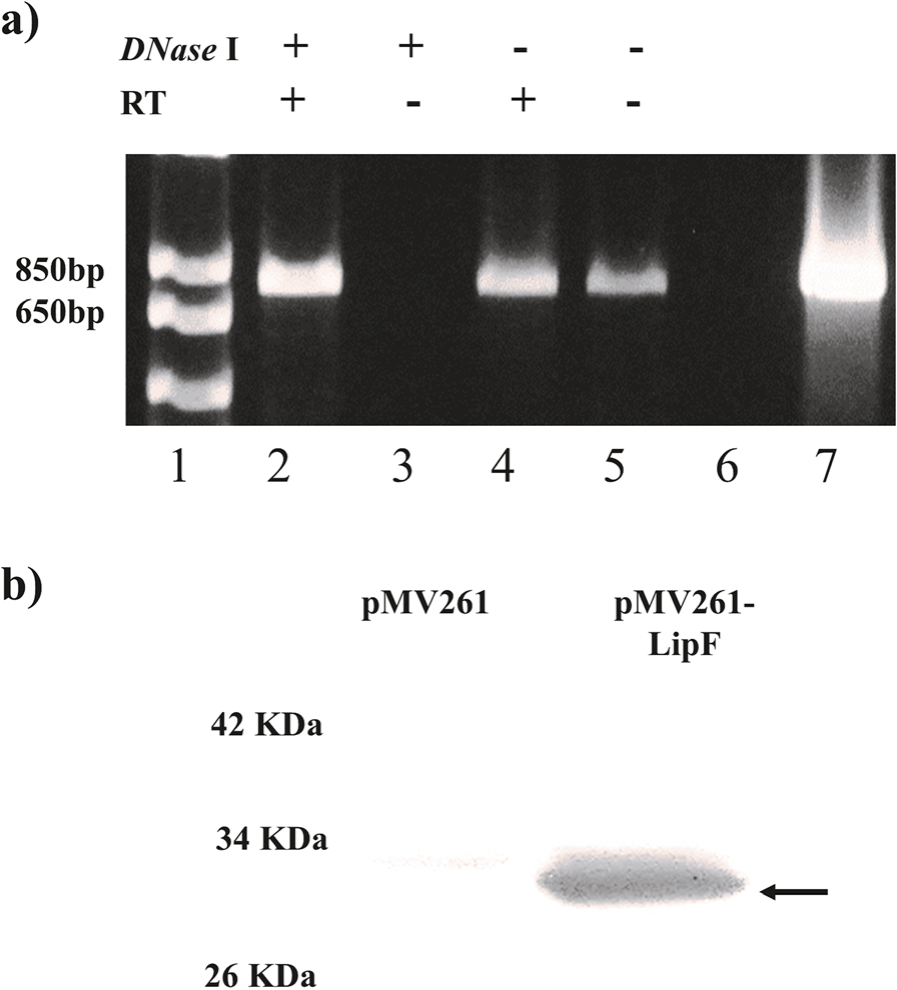
Ectopic expression of LipF in *M. smegmatis*. **a** Total RNA was extracted from pMV261-*lipF* transformants and RT-PCR assays were performed to confirm *lipF* expression in *M. smegmatis*. Lane 1 MW: 1 Kb plus DNA ladder. Lane 2–5: treatment with or without *DNase* and RT. Lane 6: negative control nuclease free water. Lane 7: positive control (pMV261-*lipF* DNA). **b** LipF protein (arrow) in *M. smegmatis* transformed with pMV261-*lipF*

To elucidate the role of Lipase F in drug resistance, we evaluated the effects of overexpressing it in a *M. tuberculosis* surrogate, *M. smegmatis*. A pMV261-*lipF* containing a specific mycobacterial control region fused to the *lipF* coding sequence was constructed. Automated Sanger sequencing verified the fidelity of sequence (data not shown), which was confirmed to have no nucleotide alterations. Following separate transformation of pMV261 or pMV261-*lipF* into *M. smegmatis* (mc^2^155 strain), reverse-transcriptase (RT)-polymerase chain reaction (PCR) analysis performed with *lipF*-specific primers confirmed that the *lipF* product (834 bp) was amplified in pMV261-*lipF* transformants. The pMV261-*lipF* transformants samples (Fig. 2 a) were treated with *DNase* I to eliminate bacterial genomic, and plasmidic DNA and RT were omitted in a control group to demonstrate that amplification was obtained solely from RNA (Fig. 2a, lanes 2 and 3). Western blot analysis with anti-LipF polyclonal antibody (see Materials and methods) confirmed the expression of a 29-kDa-*lipF* protein product in *M. smegmatis* transformed with pMV261-*lipF*, but not in pMV261-transformed controls (Fig. 2 b).

**Fig. 3:**
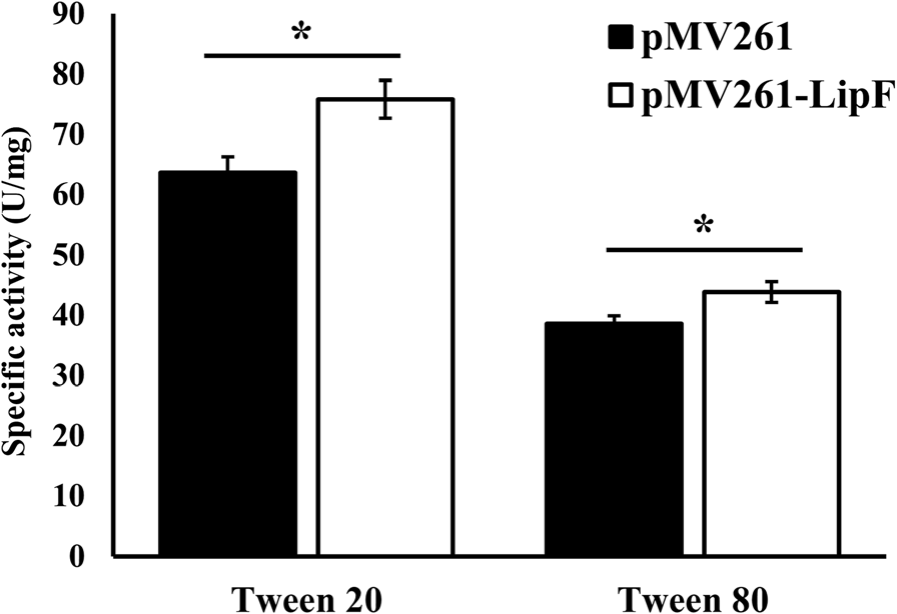
Enzymatic activity of LipF in *M. smegmatis*. Lipolytic activity assessed with cleavage of polyoxyethylene sorbitan monolaurate (Tween 20) and polyoxyethylene sorbitan monooleate (Tween 80) at pH 7.5. Assays were performed in triplicate. Error bars show standard errors, * *p ≤* 0.05

Tween cleavage assays of enzyme activity were employed to probe functionality of LipF produced from pMV261-*lipF* transformants. We observed an ~ 20% increase in esterase activity (as indexed by Tween 20 hydrolysis) with protein extract from pMV261-*lipF* transformants compared with that from pMV261 control transformants not expressing LipF. Meanwhile we found a 14% increase in lipase activity (as indexing by Tween 80 hydrolysis) in LipF expressing transformants compared with that from control pMV261 *M. smegmatis* (Fig. 3). These results demonstrate that the recombinant LipF *M. smegmatis* has increased esterase and lipase activities.

### Ectopic LipF expression modifies RIF and STR sensitivity in *M. smegmatis*

**Fig. 4:**
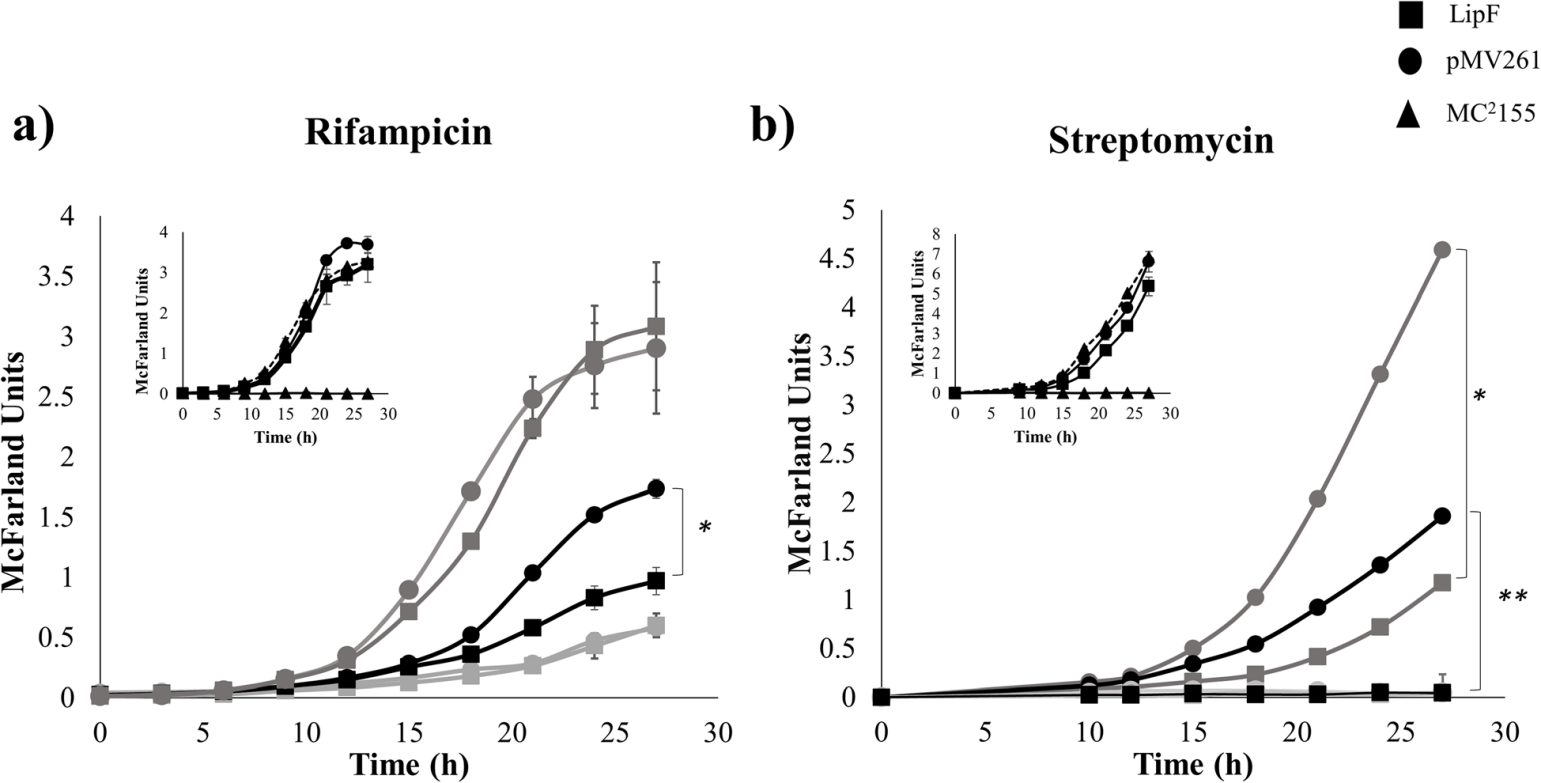
Growth kinetics of *M. smegmatis* expressing LipF in the presence of RIF and STR. Squares represent LipF-expressing *M. smegmatis*, circles represent *M. smegmatis-*pMV261 control, and triangles represent wild-type *M. smegmatis* mc^2^155 strain. **a** Light gray lines correspond to bacterial growth in medium with 3.12 μg/mL RIF; black lines correspond to growth in medium with 1.6 μg/mL RIF; and dark gray lines correspond to growth in medium with 0.8 μg/mL RIF. **b** Light gray lines correspond to bacterial growth in medium with 0.25 μg/mL; STR black lines correspond to growth in medium with 0.12 μg/mL STR; and dark gray lines correspond to growth in medium with 0.06 μg/mL STR. Growth kinetics were determined in 7H9 medium supplemented with 10% ADC, 20 μg/mL kanamycin and RIF or STR at the indicated concentrations. Growth kinetics of *M. smegmatis* expressing LipF and its control (*M. smegmatis-*pMV261) are shown in the upper left of each drug. Solid lines represent 7H9 medium supplemented with 10% ADC and 20 μg/mL kanamycin (selection antibiotic) and discontinuous lines represent medium without kanamycin. Assays were performed in duplicate. Errors bars show standard erros, *, *p* ≤ 0.05 and ** *p* ≤ 0.01

To evaluate the involvement of LipF expression in first-line TB drug resistance phenomena, we determined minimum inhibitory concentrations (MICs) for multiple antibiotic agents in a resazurin microtiter assay plate (REMA). The pMV261 and pMV261-*lipF* transformants had no changes in MICs for INH and EMB (32.0 μg/mL for INH and 0.5 μg/mL for EMB). Both transformants grew in presence of pyrazinamide (PZA) at all tested concentration (maximum 200 μg/mL) (Table 1); PZA MIC for *M. smegmatis* has previously been shown to be > 2000 μg/mL [22]. However, pMV261-*lipF* transformant was more susceptible to RIF (1.6 μg/mL) than control transformant pMV261 (3.12 μg/mL), and STR (0.06 μg/mL) versus control pMV261 (0.25 μg/mL) (Table 1). Consistent with the aforementioned MIC results, bacterial growth kinetics assays with three RIF concentrations (0.8 μg/mL, 1.6 μg/mL and 3.2 μg/mL) showed that growth was similar between the transformants pMV261 and pMV261-*lipF M. smegmatis* in presence of 3.2 μg/mL and 0.8 μg/mL RIF in 7H9 medium (Fig. 4a). However, in presence of 1.6 μg/mL RIF we observed significantly reduced growth for pMV261-LipF compared to the observed for the pMV261 control (*p* < 0.05) (Fig. 4a). Regards to STR, bacterial growth kinetics with three different STR concentrations (0.06 μg/mL, 0.12 μg/mL and 0.25 μg/mL) showed a significant growth reduction in transformants pMV261- *lipF M. smegmatis* in presence of 0.06 and 0.12 μg/mL compared with the pMV261 control (*p* < 0.05 and *p* < 0.01, respectively; Fig. 4b).

**Table 1:** MICs of first-line TB drugs in *M. smegmatis* transformants

Drug	Minimum inhibitory concentration (MIC)	
	*M. smegmatis* pMV261	*M. smegmatis* pMV261-LipF
Rifampicin	3.12 μg/mL	1.6 μg/mL
Isoniazid	32.0 μg/mL	32.0 μg/mL
Ethambutol	0.5 μg/mL	0.5 μg/mL
Pyrazinamide	> 200 μg/mL	> 200 μg/mL
Streptomycin	0.25 μg/mL	0.06 μg/mL

## Discussion

In a previous study, LipF expression was diminished in a clinical MDR *M. tuberculosis* isolate relative to that of in a pansensitive H37Rv strain. To define if this differential expression is due to mutations, we examine the regulatory regions of *lipF*, including the complete promoter regions that lies − 147 to − 623 nucleotides upstream of the translational start site of *lipF,* as well as *lipF* complete coding region from + 1 to + 834 and the intergenic region. The 477 base pair region of the promoter includes a 59 base pair minimal promoter locus responsible differential *lipF* expression under acid stress [20, 21]. We did not uncover any sequence differences between the promoters in a clinical MDR *M. tuberculosis* isolate and in the pansensitive *M. tuberculosis* reference strain. This result suggests that other mechanisms are involved in the differential expression of LipF in MDR *M. tuberculosis*.

We selected *M. smegmatis* as a surrogate species for *M. tuberculosis* because it can be handled safely, and it grows fast. Heterologous expression of LipF in *M. smegmatis* was confirmed with the affirmation of the presence of *lipF* transcript and an associated protein of the expected molecular weight for LipF (29 kDa) [15]. The functionality of this protein was analyzed in Tween-cleavage enzyme assays. Tween cleavage assays which have been used extensively to determine lipase/esterase activity, have been reported to be 36 times more sensitive than the titrimetric assay (using triacetic acids) and four times more sensitive than spectrophotometry with *p-*NP palmitate [17, 23–25]. Our finding of increased esterase activity in the LipF-expressing strain is consistent with prior work demonstrating esterase activity for LipF, which prefers water-soluble short chain fatty acid esters as substrates and has a high affinity for glycerol acetate and *p-*NP acetate. LipF has not been shown previously to act on triacylglycerides or *p-*NP esters with long-chain fatty acids, such as tricaprin and *p-*NP caprate [15]. Notably, we found a 14% increase in lipase activity acting on long-chain fatty acids. Therefore, the present data confirmed lipase and esterase activities of the recombinant LipF.

Determination of MICs for bacteria is a gold standard method in antimicrobial susceptibility wherein a MIC is defined as the lowest concentration inhibits detectable growth of a microorganism over predetermined time period [26]. The presently determined MICs for wild-type *M. smegmatis* were different from previously reported MICs [27]. This discordance could be due to the use of parafilm to seal plates for 40 h incubation in previous assays; sealing may reduce oxygen availability, which could slow growth of mycobacteria and increase their susceptibility to drugs. Regardless, the MICs obtained in the present study were reproducible. Compared to controls, *M. smegmatis* transformants expressing LipF had a reduced RIF and STR MICs but unaltered EMB and INH MICs. Consistent with our RIF and STR MICs results, a bacterial growth assay showed slower growth in LipF-expressing transformants cultured in medium containing 1.6 μg/mL RIF (*p* < 0.05), and in cultures in medium containing 0.12 μg/mL and 0.06 μg/mL STR (*p <* 0.01 and *p <* 0.05, respectively). These results indicate that LipF expression in *M. smegmatis* confers an enhance sensitivity to RIF and STR.

Previously, Rv0183, LipX, and LipG lipases, these last from the Lip family, have been related to drug resistance. Disruption of a Rv0183 homolog in *M. smegmatis* modified sensitivity to RIF and INH [28]. LipX overexpression in *M. smegmatis* was shown to increase resistance to the first-line anti-TB drugs INH, EMB, and RIF, whereas LipG disruption in *M. smegmatis* was shown to augment RIF and INH resistance [17, 18]. It is unclear whether enhance RIF sensitivity in *M. smegmatis* expressing LipF in our study can be attribute to a direct interaction between RIF and LipF. A molecular docking experiment show only one possible mode of interaction, which was weak (hydrogen bond with a root-mean-square deviation value < 3) (data not shown). Meanwhile LipX and LipG which can modify mycobacterial drug resistance alter the composition of cell walls by increasing their glycopeptidelipid contents [17, 18]. Being a non-polar molecule, RIF can enter the cell through passive diffusion [29]. Interestingly, a RIF persistent *M. tuberculosis* strain was recently reported to have a thick outer layer, atypically abundant polysaccharides, whose presence increases hydrophobicity and reduces efflux pumps-independent RIF influx [30]. Thus, changes in cell wall composition and polarity can alter the access of antibiotic agents, such as RIF, to the inside of bacteria. Resistance to aminoglycosides has been associated to bacterial cell membranes alterations regarding permeability [31]; a diffusion of STR when porins were inhibited in *E. coli* have been demonstrated [32]. Moreover some *M. tuberculosis* clinical isolates resistant to STR has been associated with a lower cell wall permeability [33]. Given this potential, we suggest that the esterase and lipase activities of LipF could, potentially increase mycobacterial susceptibility to RIF and STR by modifying mycobacterial cell walls in a manner that enable entry of these drugs into cell.

Overexpression of *rpoB* has been implicated in RIF resistance in *Mycobacterium* [34]. In order to demonstrate if *rpoB* expression is involved in rifampicin and streptomycin susceptibility we performed an expression analysis using a RT-PCR assay and a semi-quantitative densitometric analysis and we did not find differences in expression of *rpoB* gene between strains expressing LipF and its empty vector control (data no shown).

Although inhibition of *lipF* has been shown to modify *M. tuberculosis* growth in a previous study [35]; we did not observe effects of LipF expression on *M. smegmatis* growth in this study (Fig. S1). Rather, we found that, ectopic expression of LipF increased *M. smegmatis* sensitivity to RIF and STR suggesting that reduced-lipF expression may contribute to the development of RIF and STR resistance in mycobacteria. More studies are needed to demonstrate LipF participation in drug resistance, to clarify the mechanism by which LipF may contribute to RIF and STR resistance. Furthermore, more analyses with second line drugs are needed to identify other potential roles of lipases in drug resistance.

## Conclusions

In conclusion, we demonstrated that ectopic expression of LipF increases sensitivity to RIF and STR. These results suggest that low LipF expression in *M. tuberculosis* may be an important enabler for RIF and STR resistance.

## Methods

### Strains and growth conditions

DH5α strain *Escherichia coli (E. coli)* was used for construct transformations, and mc^2^155 *M. smegmatis* strain was used as a protein expression surrogate for *M. tuberculosis*. H37Rv *strain M. tuberculosis* (GenBank: AL123456.3) and the clinical CIBIN:UMF:15:99 MDR *M. tuberculosis* isolate were obtained from a repository located in the Centro de Investigación Biomédica del Noreste, Instituto Mexicano del Seguro Social.

*E. coli* was grown in LB medium supplemented with 75 μg/mL kanamycin for transformant selection, liquid or solid agar. *M. smegmatis* was grown in Middlebrook 7H9 medium supplemented with 10% album-dextrose-catalase (ADC) and 0.05% Tween 80; 20 μg/mL kanamycin was added for transformant selection. *E. coli* and *M. smegmatis* were incubated at 37 °C for 24 h for liquid cultures and 3 d for solid cultures. *M. tuberculosis* was grown as previously reported [14]. Briefly, Middlebrook 7H9 medium supplemented with 10% oleic acid, albumin, dextrose, and catalase (OADC). These cultures were incubated at 37 °C in a 5% CO_2_ atmosphere until they reached a turbidity equivalent to the 1.0 McFarland standard. A 100 μL aliquot was inoculated in 10 mL of the aforementioned supplemented Middlebrook 7H9 medium. Liquid cultures were incubated at 37 °C in a 5% CO_2_ atmosphere with constant orbital shaking (300 rpm) until they reached log phase growth (i.e., 3.0–4.0 McFarland).

For growth curves, pMV261 and pMV261-*lipF* transformants of *M. smegmatis* were grown until they reached 1.0 McFarland unit. Then, they were re-inoculated in fresh medium (3.08 × 10^5^ bacteria/mL) with RIF (0.8 μg/mL, 1.6 μg/mL and 3.12 μg/mL) or STR (0.25 μg/mL, 0.12 μg/mL and 0.06 μg/mL) and 20 μg/mL kanamycin. Cultures were incubated at 37 °C with constant shaking for 27 h, and McFarland units were measured every 3 h with a DensiCHECK™Plus nephelometer (BioMérieux, Marcy-l’Étoile, France). All experiments were performed in duplicate, and average values were used to generate the growth kinetic curves.

### MIC determination

MICs for the first line TB drugs (rifampicin, isoniazid, ethambutol and pyrazinamide) and STR were determined with REMA method, previously described [27]. Briefly, 96-well plates were filled with 50 μL of 7H9 media (for PZA the medium was acidified at pH 5.5) except for the first column. Antibiotic concentration stocks were prepared at double the needed concentrations and 100-μL were added to each well of the first column, and then diluted serially by half concentration by mixing with media in the subsequent wells. The last columns were used as no-antibiotic controls. Plates were inoculated with 2.77 × 10^4^ bacteria/mL and incubated at 37 °C under constant agitation for 40 h. Subsecuently, 30 μL of resazurin reagent (20 μg/mL) was added to each well and incubated for 6 h. MICs were then determined when a change of color from blue to pink was observed.

### Plasmid construction

Genomic DNA from *M. tuberculosis* (H37Rv strain) was used as an amplification template. To amplify an 834-base pair product, the following specific primers for the *lipF* coding sequence were used: forward 5′-CAC CGG ATC CAA TGC GTG CGC CTG GGG TG-3′; and reverse 5′-GGA TCC CTA GAT AGG CGA CCT GTC CAA AC-3′ (underlined sequence corresponds to *Bam*HI recognition site). PCR was performed in a final volume of 25 μL with the following constituents: *Taq* DNA polymerase buffer 1×, 2 mM dNTPs, 3.5 mM MgCl_2_, 5% DMSO, and 1 U *Taq* DNA. The PCR program was: one 5-min denaturation period 95 °C, thirty 30s cycles of denaturation at 95 °C, primer annealing at 58 °C for 30 s, polymerization at 72 °C for 30 s, and a final 5 min step at 72 °C. The PCR products were visualized by electrophoresis in a 1% (w/v) agarose gel stained with Gel red (Biotium, Hayward, USA). The *lipF* PCR product was inserted in pET101/D plasmid (Invitrogen, USA), as a transition vector.

For *lipF* expression in *M. smegmatis*, the mycobacterial pMV261 vector was used. The pET101/D-LipF construct and pMV261 vector were digested with *Bam*HI enzyme, and ligation of *lipF* in pMV261 was performed with T4 DNA ligase (Invitrogen, Carlsbad, CA). Positive clones, defined as having the gene inserted in the sense direction in pMV261-*lipF,* were confirmed by PCR amplification with a primer that aligns in a vector region (pMV261 Forward 5′-GTTGTAGTGCTTGTGTGGCA-3′) and the *lipF* reverse primer and DNA sequencing.

### RNA and protein expression analysis

*M. smegmatis* mc^2^155 cells were transformed by electroporation with pMV261 or pMV261-*lipF* construct and incubated at 37 °C on 7H10 agar plates containing 20 μg/mL kanamycin. After 3 d of incubation, single colonies were isolated and grown in 10 mL of 7H9 supplemented with 10% ADC, 0.05% Tween 80 and 20 μg/mL kanamycin. The culture conditions were 37 °C with of constant shaking at 300 rpm for 20 h. Then, total RNA was extracted with TRIzol reagent (Invitrogen, Carlsbad, CA) according to manufacturer’s instructions. Briefly, 10 mL of mycobacterial culture was centrifuged for 20 min at 25,000×g at 4 °C; after supernatant removal, the bacterial pellet was suspended in 1 mL of TRIzol reagent and incubated for 20 min at room temperature. Samples were then transferred to Fast Prep tubes containing Lysin Matrix B (MP Biomedicals, Solon, OH. USA) and processed for three 20 s cycles at a velocity setting of 6, with 3 min cooling on ice period between cycles. RNA was precipitated with chloroform and isopropyl alcohol and then, washed with ethanol. Finally, RNA was resuspended in Tris-EDTA buffer (Promega, Madison, WI. USA) and treated with *DNase* I (Invitrogen, Carlsbad, CA. USA).

The purity and concentration of RNA were estimated by a Nanodrop spectrophotometer. Synthesis of cDNA was carried out with M-MLV RT in the presence of random primers (Invitrogen, Carlsbad, CA. USA) following the conditions specified by the manufacturer. RT reaction mix 1 (0.6–2 μg of total RNA, random primers and deoxynucleoside triphosphate) was incubated at 65 °C for 5 min and then cooled on ice. Reaction mix 2 (first strand buffer 1×, DTT and RNase OUT) was added to mix 1 and incubated at 37 °C for 2 min. Then M-MLV RT was added. The final reaction was incubated at 25 °C for 10 min, followed by 37 °C for 50 min, and then 70 °C for 15 min.

For western blotting, specific antibodies against LipF were designed (Genemed Synthesis, Inc., San Antonio, TX). For protein extraction, frozen bacteria stocks were inoculated in 3 mL of 7H9 media supplemented with 10% ADC, 0.05% Tween 80 and 20 μg/mL kanamycin. After 20 h, an inoculum of 100 μL was transferred into 30 mL of 7H9 medium. The cultures were incubated at 37 °C for 20 h, centrifuged, and the bacteria pellets were washed in 1× phosphate buffered saline to eliminate albumin residue. Bacterial lysis was performed with a FastPrep instrument (MP Biomedicals, Solon, OH.USA), wherein bacteria pellets were dissolved in 500 μL of lysis buffer (150 mM NaCl, 1 mM β-mercaptoethanol, pH 8.15 and proteases inhibitors) and transferred to Fast Prep tubes containing Matrix B (MP Biomedicals, Solon, OH. USA). Cell lysis was obtained using three 20 s cycles at a velocity setting of 6, and samples were cooled on ice between each cycle. The supernatant was collected after centrifugation at 15,294×g (5430R Eppendorf) for 3 min and stored to − 20 °C until use.

### Enzymatic activity

*M. smegmatis* pMV261 and *M. smegmatis* pMV261-*lipF* were grown in 20 mL of 7H9 medium supplemented with 10% ADC, 0.05% Tween 80 and 20 μg/mL kanamycin until the cultures reached 4.0 McFarland units. Cultures were centrifuged, washed and resuspended in 800 μL of lysis buffer as described previously [17] (50 mM Tris-Cl of pH 8.0, 300 mM NaCl and protease inhibitors). Bacteria lysis was performed as mentioned above and protein concentration was determined by the Bradford method (Biorad, CA, USA).

Lipolytic activity was determined as described previously [17]. Briefly, a reaction mixture was prepared in 50 mM Tris, 33 mM CaCl_2,_ 0.33% Tween 80 or Tween 20 [pH 7.5] and 40 μg cell lysate. This reaction mixture was incubated at 35 °C for 30 min. When the incubation was finished, 200 μL were transferred to a 96-well microwell plate (Corning, NY, USA), and turbidity was assessed by measuring absorbance at 405 nm. Enzymatic activity was reported as U/mg of lysate, where 1 U is defined as the amount of enzyme required to induce a change in absorbance of 0.01 under the assay conditions. Assays were performed in triplicate.

### Sequencing and analysis

Mycobacterial DNA was isolated by the cetyltrimethylammonium bromide (CTAB) method as described in Peñuelas-Urquides et al. (2013) [14]. Automated Sanger sequencing was performed with *M. tuberculosis* H37Rv and CIBIN:UMF:15:99 genomic DNA. The purified PCR products were sequenced with a BigDye™ Terminator v3.1 Cycle Sequencing Kit following the manufacturer’s instruction in a 3130xl Genetic Analyzer (Applied Biosystems, Foster City, CA, USA) with the following primers: LipF promoter, forward 5′-CCG TGC AAA TAG AGC ACC AC-3′ (LipF Prom-F); LipF internal forward (LipF_Int-F), 5′-AAA CGT GAA TAA GTG TCG GC-3′, and LipF internal reverse (LipF_Int-R) 5′- CAG TGC GAC GAC GAG AAA-3′(Fig. 1). The sequence analysis was performed Seqscape v2.7 software with the GenBank reference NC_000962.3.

### Statistics

Growth curves were developed based on biological duplicates, and statistical analyses were performed through slope of the line comparisons. Enzymatic activity was determined in technical triplicates assays and analyzed with Mann-Whitney tests in SPSS v.20 (IBM). *P* values ≤0.05 were considered significant.

## Supplementary information

**Additional file 1. **LipF sequence comparison between *M. tuberculosis* H37Rv and clinical CIBIN:UMF:15:99. H37Rv isolate. GenBank: CP009480.1 was used for reference. The LipF coding sequence is underlined, and the LipF promoter sequence is marked in gray. Sequences were obtained through automated sequencing and aligned in CLC sequence viewer 8.0 software.

**Additional file 2: Fig. S1.** Growth kinetics of *M. smegmatis* expressing LipF in a) RIF and b) STR assays. Growth of strains represent the control in medium without RIF and STR. Solid lines represent 7H9 medium supplemented with 10% ADC and 20 μg/mL kanamycin (selection antibiotic) and discontinuous lines represent medium without kanamycin. Squares represent LipF-expressing *M. smegmatis*, circles represent *M. smegmatis-*pMV261 control, and triangles represent wild-type *M. smegmatis* mc^2^155 strain.

## Data Availability

Please contact corresponding author for data request.

## Abbreviations

TB: Tuberculosis
STR: Streptomycin
EMB: Ethambutol
RIF: Rifampicin
INH: Isoniazid
PZA: Pyrazinamide
MDR: Multidrug resistance
ADC: Albumin, dextrose and catalase
OADC: Oleic acid, albumin, dextrose and catalase
MIC: Minimal inhibitory concentration
REMA: Resazurin microtiter assay plate
pNP: *p-*Nitrophenyl
