# Supplementary material for: LipF increases rifampicin and streptomycin sensitivity in a Mycobacterium tuberculosis surrogate
Source: BMC Microbiol. 2020 May 25;20:132. doi: 10.1186/s12866-020-01802-x (PMC7249682; doi:10.1186/s12866-020-01802-x)
Supplement: Supplementary file 1 — Additional file 1. LipF sequence comparison between M. tuberculosis H37Rv and clinical CIBIN:UMF:15:99. H37Rv isolate. GenBank: CP009480.1 was used for reference. The LipF coding sequence is underlined, and the LipF promoter sequence is marked in gray. Sequences were obtained through automated sequencing and aligned in CLC sequence viewer 8.0 software. [file 12866_2020_1802_MOESM1_ESM.pdf]

```
                                                         20                                      40
CP009480.1:H37Rv_LipFgene  -------CTA GATAGGCGAC TGTCCAAACG CGCCACGGCT TGCGGCGCGA 43
                    H37Rv  TGGATCCCTA GATAGGCGAC TGTCCAAACG CGCCACGGCT TGCGGCGCGA 50
           CIBIN:UMF:15:99 -------CTA GATAGGCGAC TGTCCAAACG CGCCACGGCT TGCGGCGCGA 43
                                     60                    80                    100
CP009480.1:H37Rv_LipFgene  GGTGAACCCG CGACGTAGCG CGACCGATGC ACCGGACTCA ACGACGAGTC 93
                    H37Rv  GGTGAACCCG CGACGTAGCG CGACCGATGC ACCGGACTCA ACGACGAGTC 100
           CIBIN:UMF:15:99 GGTGAACCCG CGACGTAGCG CGACCGATGC ACCGGACTCA ACGACGAGTC 93
                                                120                   140
CP009480.1:H37Rv_LipFgene  AGCGGTGGCG TCGCGAATGA ACTGCCCGAT CTGACGCAAC GAACGGGTCG 143
                    H37Rv  AGCGGTGGCG TCGCGAATGA ACTGCCCGAT CTGACGCAAC GAACGGGTCG 150
           CIBIN:UMF:15:99 AGCGGTGGCG TCGCGAATGA ACTGCCCGAT CTGACGCAAC GAACGGGTCG 143
                                    160                   180                   200
CP009480.1:H37Rv_LipFgene  CTTCGGGCAC CAGCGGTGTG GCGAGTTGGA AAAGATGAGC CTGACCGGGC 193
                    H37Rv  CTTCGGGCAC CAGCGGTGTG GCGAGTTGGA AAAGATGAGC CTGACCGGGC 200
           CIBIN:UMF:15:99 CTTCGGGCAC CAGCGGTGTG GCGAGTTGGA AAAGATGAGC CTGACCGGGC 193
                                                220                   240
CP009480.1:H37Rv_LipFgene  CAAACCCGTA CCTCGGCACA GACGCCTGCC GCCGCCAGCT TGCCGGCGCC 243
                    H37Rv  CAAACCCGTA CCTCGGCACA GACGCCTGCC GCCGCCAGCT TGCCGGCGCC 250
           CIBIN:UMF:15:99 CAAACCCGTA CCTCGGCACA GACGCCTGCC GCCGCCAGCT TGCCGGCGCC 243
                                    260                   280                   300
CP009480.1:H37Rv_LipFgene  CAGCTGCGCG TCGTGCAGCA GCACTTCGGA GCCGGAAACG TGAATAAGTG 293
                    H37Rv  CAGCTGCGCG TCGTGCAGCA GCACTTCGGA GCCGGAAACG TGAATAAGTG 300
           CIBIN:UMF:15:99 CAGCTGCGCG TCGTGCAGCA GCACTTCGGA GCCGGAAACG TGAATAAGTG 293
                                                320                   340
CP009480.1:H37Rv_LipFgene  TCGGCGGCAA GCTGGATTCG ATATGGTCGA GCGGCTCATA GAGGTCTTCG 343
                    H37Rv  TCGGCGGCAA GCTGGATTCG ATATGGTCGA GCGGCTCATA GAGGTCTTCG 350
           CIBIN:UMF:15:99 TCGGCGGCAA GCTGGATTAC GAATGGTCGA GCGGCTCATA GAGGTCTTCG 343
                                    360                   380                   400
CP009480.1:H37Rv_LipFgene  GGCCTGCCGT CGACCATGTT CTTGGCAGCG GCCGCCCTGA CCCATGCCGC 393
                    H37Rv  GGCCTGCCGT CGACCATGTT CTTGGCAGCG GCCGCCCTGA CCCATGCCGC 400
           CIBIN:UMF:15:99 GGCCTGCCGT CGACCATGTT CTTGGCAGCG GCCGCCCTGA CCCATGCCGC 393
                                                420                   440
CP009480.1:H37Rv_LipFgene  CAAGGCATCG AATGCCCGCG CCGGAAACAT CGCGTCGGTC CCGATGTTGG 443
                    H37Rv  CAAGGCATCG AATGCCCGCG CCGGAAACAT CGCGTCGGTC CCGATGTTGG 450
           CIBIN:UMF:15:99 CAAGGCATCG AATGCCCGCG CCGGAAACAT CGCGTCGGTC CCGATGTTGG 443
                                    460                   480                   500
CP009480.1:H37Rv_LipFgene  GATGGTCCTG CTTGGGCCCC TTGGCCAGCT GCAGCAACGG AGAGATGGCC 493
                    H37Rv  GATGGTCCTG CTTGGGCCCC TTGGCCAGCT GCAGCAACGG AGAGATGGCC 500
           CIBIN:UMF:15:99 GATGGTCCTG CTTGGGCCCC TTGGCCAGCT GCAGCAACGG AGAGATGGCC 493
                                                520                   540
CP009480.1:H37Rv_LipFgene  ACTATTGCCG CCGGTTTCTC GTCGTCGCAC TGCAGCCGCT GCGCAAGCGC 543
                    H37Rv  ACTATTGCCG CCGGTTTCTC GTCGTCGCAC TGCAGCCGCT GCGCAAGCGC 550
           CIBIN:UMF:15:99 ACTATTGCCG CCGGTTTCTC GTCGTCGCAC TGCAGCCGCT GCGCAAGCGC 543
                                    560                   580                   600
CP009480.1:H37Rv_LipFgene  GAGCGCAAGG TAACCACCCG CGGAATCACC GGCCAACACG ATCTGTTCCG 593
                    H37Rv  GAGCGCAAGG TAACCACCCG CGGAATCACC GGCCAACACG ATCTGTTCCG 600
           CIBIN:UMF:15:99 GAGCGCAAGG TAACCACCCG CGGAATCACC GGCCAACACG ATCTGTTCCG 593
                                                620                   640
CP009480.1:H37Rv_LipFgene  GCCGGTATCC GCGCGCCCGC AACCATTGGT ATGCATCGTG GCAGTCGTCG 643
                    H37Rv  GCCGGTATCC GCGCGCCCGC AACCATTGGT ATGCATCGTG GCAGTCGTCG 650
           CIBIN:UMF:15:99 GCCGGTATCC GCGCGCCCGC AACCATTGGT ATGCATCGTG GCAGTCGTCG 643
```

```
                                      660                                680                                700
                                       |                                  |                                  |
CP009480.1:H37Rv_LipFgene  AGCGCCATCC  CCAGCGAATG  CTTAGGGATC  AGCCGATAGT  CGACTATCAA  693
                    H37Rv  AGCGCCATCC  CCAGCGAATG  CTTAGGGATC  AGCCGATAGT  CGACTATCAA  700
          CIBIN:UMF:15:99  AGCGCCATCC  CCAGCGAATG  CTTAGGGATC  AGCCGATAGT  CGACTATCAA  693

                                                   720                                740
                                                    |                                  |
CP009480.1:H37Rv_LipFgene  CACGGGTGAT  TCGGCAAATC  CTGACAGCGC  GTTGACGATC  CTGCTGTGCG  743
                    H37Rv  CACGGGTGAT  TCGGCAAATC  CTGACAGCGC  GTTGACGATC  CTGCTGTGCG  750
          CIBIN:UMF:15:99  CACGGGTGAT  TCGGCAAATC  CTGACAGCGC  GTTGACGATC  CTGCTGTGCG  743

                                      760                                780                                800
                                       |                                  |                                  |
CP009480.1:H37Rv_LipFgene  AATTCGGCCC  GCACATGACA  AACGCGCCGC  CGTGCAAATA  GAGCACCACC  793
                    H37Rv  AATTCGGCCC  GCACATGACA  AACGCGCCGC  CGTGCAAATA  GAGCACCACC  800
          CIBIN:UMF:15:99  AATTCGGCCC  GCACATGACA  AACGCGCCGC  CGTGCAAATA  GAGCACCACC  793

                                                   820                                840
                                                    |                                  |
CP009480.1:H37Rv_LipFgene  CGCCCAGCGC  CGTCGGCCGC  CCGCACCCCA  GGCGCACGCA  CCAACTGGGC  843
                    H37Rv  CGCCCAGCGC  CGTCGGCCGC  CCGCACCCCA  GGCGCACGCA  CCAACTGGGC  850
          CIBIN:UMF:15:99  CGCCCAGCGC  CGTCGGCCGC  CCGCACCCCA  GGCGCACGCA  CCAACTGGGC  843

                                      860                                880                                900
                                       |                                  |                                  |
CP009480.1:H37Rv_LipFgene  GGTAGCATTC  GGCAAATTTA  TCGTTGTTCG  GACCGTGCCC  TGCCCGGGGC  893
                    H37Rv  GGTAGCATTC  GGCAAATTTA  TCGTTGTTCG  GACCGTGCCC  TGCCCGGGGC  900
          CIBIN:UMF:15:99  GGTAGCATTC  GGCAAATTTA  TCGTTGTTCG  GACCGTGCCC  TGCCCGGGGC  893

                                                   920                                940
                                                    |                                  |
CP009480.1:H37Rv_LipFgene  GCCAAACCCT  GCATGCGAAG  TCGACGAACC  CCAACGGCAG  AGGCAGGGGC  943
                    H37Rv  GCCAAACCCT  GCATGCGAAG  TCGACGAACC  CCAACGGCAG  AGGCAGGGGC  950
          CIBIN:UMF:15:99  GCCAAACCCT  GCATGCGAAG  TCGACGAACC  CCAACGGCAG  AGGCAGGGGC  943

                                      960                                980                              1,000
                                       |                                  |                                  |
CP009480.1:H37Rv_LipFgene  GATAGGTAAC  TGCCCACAGT  CATAAGTGGC  TTGATCGTCA  TGCGCGATGC  993
                    H37Rv  GATAGGTAAC  TGCCCACAGT  CATAAGTGGC  TTGATCGTCA  TGCGCGATGC  1000
          CIBIN:UMF:15:99  GATAGGTAAC  TGCCCACAGT  CATAAGTGGC  TTGATCGTCA  TGCGCGATGC  993

                                                 1,020                              1,040
                                                    |                                  |
CP009480.1:H37Rv_LipFgene  CAGTGCCGCC  AACCGACCTG  CAACACTAGG  GCCGCTTTCG  GTGATCTCGA  1043
                    H37Rv  CAGTGCCGCC  AACCGACCTG  CAACACTAGG  GCCGCTTTCG  GTGATCTCGA  1050
          CIBIN:UMF:15:99  CAGTGCCGCC  AACCGACCTG  CAACACTAGG  GCCGCTTTCG  GTGATCTCGA  1043

                                    1,060                              1,080                              1,100
                                       |                                  |                                  |
CP009480.1:H37Rv_LipFgene  TGGGAGCCCC  GTCCCAGCAC  GAATCGGAAT  TCGAGCATCC  CGACGATTGC  1093
                    H37Rv  TGGGAGCCCC  GTCCCAGCAC  GAATCGGAAT  TCGAGCATCC  CGACGATTGC  1100
          CIBIN:UMF:15:99  TGGGAGCCCC  GTCCCAGCAC  GAATCGGAAT  TCGAGCATCC  CGACGATTGC  1093

                                                 1,120                              1,140
                                                    |                                  |
CP009480.1:H37Rv_LipFgene  AGGGGCCGGC  GTGCGTAATA  CGAGGACATT  TTCAGCACGT  TTCGCCGGAA  1143
                    H37Rv  AGGGGCCGGC  GTGCGTAATA  CGAGGACATT  TTCAGCACGT  TTCGCCGGAA  1150
          CIBIN:UMF:15:99  AGGGGCCGGC  GTGCGTAATA  CGAGGACATT  TTCAGCACGT  TTCGCCGGAA  1143

                                    1,160                              1,180                              1,200
                                       |                                  |                                  |
CP009480.1:H37Rv_LipFgene  TGTGGCCGGT  GGTTGGCGTT  AGCTGCACGG  AAGCGCCTGA  GCTGGCCCGC  1193
                    H37Rv  TGTGGCCGGT  GGTTGGCGTT  AGCTGCACGG  AAGCGCCTGA  GCTGGCCCGC  1200
          CIBIN:UMF:15:99  TGTGGCCGGT  GGTTGGCGTT  AGCTGCACGG  AAGCGCCTGA  GCTGGCCCGC  1193

                                                 1,220                              1,240
                                                    |                                  |
CP009480.1:H37Rv_LipFgene  CGTCACCGCC  CGATTTATCA  ATCGCAAATC  TCGCACTTCC  CGTTTACGTA  1243
                    H37Rv  CGTCACCGCC  CGATTTATCA  ATCGCAAATC  TCGCACTTCC  CGTTTACGTA  1250
          CIBIN:UMF:15:99  CGTCACCGCC  CGATTTATCA  ATCGCAAATC  TCGCACTTCC  CGTTTACGTA  1243

                                    1,260                              1,280                              1,300
                                       |                                  |                                  |
CP009480.1:H37Rv_LipFgene  GTTGCTCCAA  CCAGACGCAG  CCCAATTCGG  GCTCCTCCCC  CCATCAATCA  1293
                    H37Rv  GTTGCTCCAA  CCAGACGCAG  CCCAATTCGG  GCTCCTCCCC  CCATCAATCA  1300
          CIBIN:UMF:15:99  GTTGCTCCAA  CCAGACGCAG  CCCAATTCGG  GCTCCTCCCC  CCATCAATCA  1293
```

```
                                                      1,320                              1,340
                                                        |                                  |
CP009480.1:H37Rv_LipFgene  TTCGGTGGCG CGAAGTTCAC CAGAGTCCCG GACACGCTCA CGCGAACTAC 1343
                    H37Rv  TTCGGTGGCG CGAAGTTCAC CAGAGTCCCG GACACGCTCA CGCGAACTAC 1350
          CIBIN:UMF:15:99  TTCGGTGGCG CGAAGTTCAC CAGAGTCCCG GACACGCTCA CGCGAACTAC 1343
                                 1,360                              1,380                            1,400
                                   |                                  |                                |
CP009480.1:H37Rv_LipFgene  CTGCATTTAG GGGATCACAG GCACCTTGAA ATGCATCGGT GTATGACTGG 1393
                    H37Rv  CTGCATTTAG GGGATCACAG GCACCTTGAA ATGCATCGGT GTATGACTGG 1400
          CIBIN:UMF:15:99  CTGCATTTAG GGGATCACAG GCACCTTGAA ATGCATCGGT GTATGACTGG 1393
                                                      1,420                              1,440
                                                        |                                  |
CP009480.1:H37Rv_LipFgene  GAGTTTGCTG TACGTCTATT GGTAAGTGCG AATTCGCCGC CGGCTACCCG 1443
                    H37Rv  GAGTTTGCTG TACGTCTATT GGTAAGTGCG AATTCGCCGC CGGCTACCCG 1450
          CIBIN:UMF:15:99  GAGTTTGCTG TACGTCTATT GGTAAGTGCG AATTCGCCGC CGGCTACCCG 1443
                                 1,460                              1,480                            1,500
                                   |                                  |                                |
CP009480.1:H37Rv_LipFgene  CACCCCGTAG AATCGCAAGC CGATATCGGC TTGGTCACCT GAGGTGTTCT 1493
                    H37Rv  CACCCCGTAG AATCG----- ---------- ---------- ---------- 1465
          CIBIN:UMF:15:99  CACCCCGTAG AATCG----- ---------- ---------- ---------- 1458
                                                      1,520
                                                        |
CP009480.1:H37Rv_LipFgene  ATGCGGGAGT TTCAGCGGGC CGCGGTGCGC CTGCACAT 1531
                    H37Rv  ---------- ---------- ---------- -------- 1465
          CIBIN:UMF:15:99  ---------- ---------- ---------- -------- 1458
```
