# Supplementary figures and images for: LipF increases rifampicin and streptomycin sensitivity in a Mycobacterium tuberculosis surrogate
Source: BMC Microbiol. 2020 May 25;20:132. doi: 10.1186/s12866-020-01802-x (PMC7249682; doi:10.1186/s12866-020-01802-x)

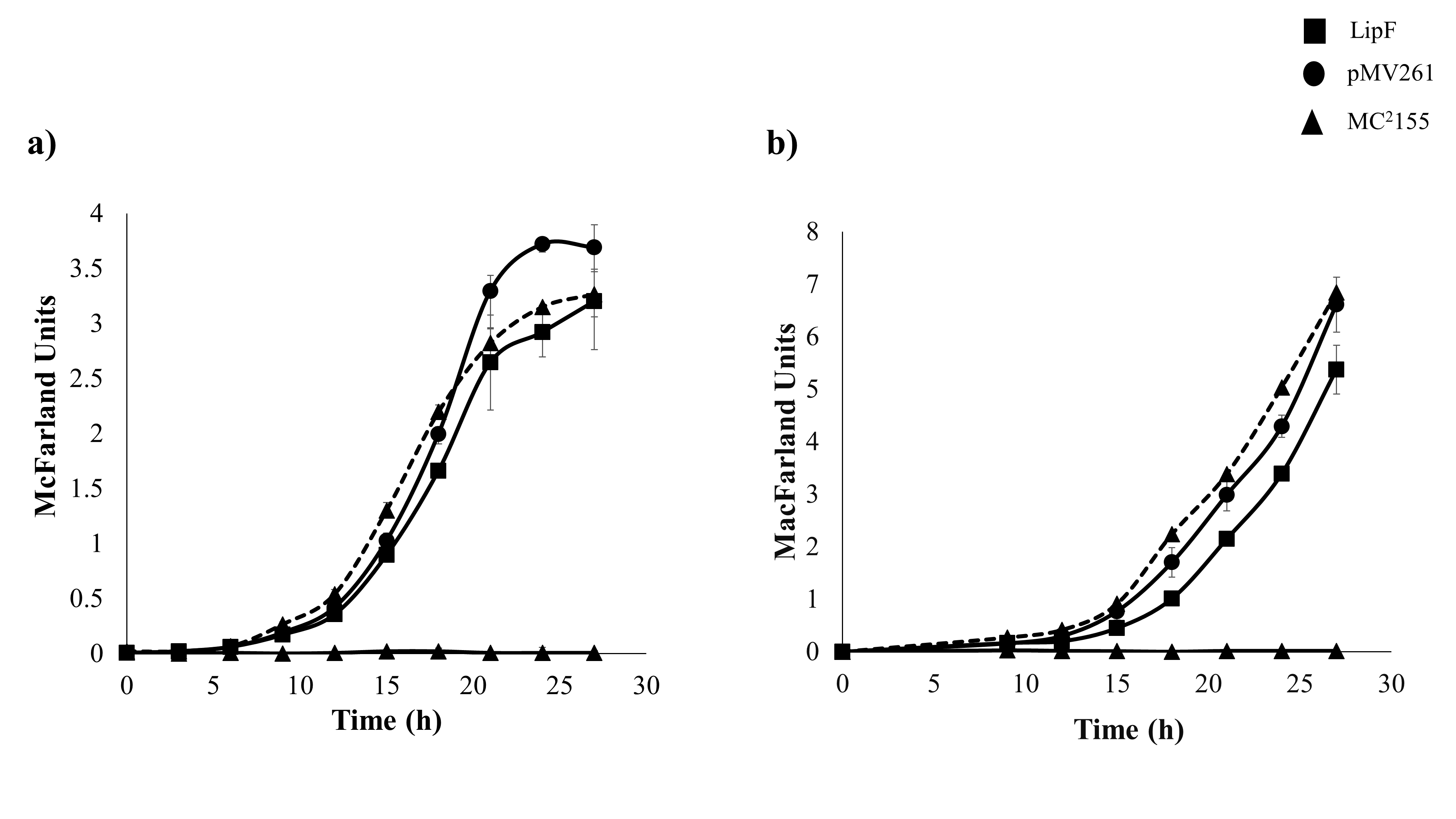

Supplement: Supplementary file 2 — Additional file 2: Fig. S1. Growth kinetics of M. smegmatis expressing LipF in a) RIF and b) STR assays. Growth of strains represent the control in medium without RIF and STR. Solid lines represent 7H9 medium supplemented with 10% ADC and 20 μg/mL kanamycin (selection antibiotic) and discontinuous lines represent medium without kanamycin. Squares represent LipF-expressing M. smegmatis, circles represent M. smegmatis-pMV261 control, and triangles represent wild-type M. smegmatis mc2155 strain. [file 12866_2020_1802_MOESM2_ESM.tif]
